# Environmental Factors Affecting Large-Bodied Coral Reef Fish Assemblages in the Mariana Archipelago

**DOI:** 10.1371/journal.pone.0031374

**Published:** 2012-02-27

**Authors:** Benjamin L. Richards, Ivor D. Williams, Oliver J. Vetter, Gareth J. Williams

**Affiliations:** 1 Department of Zoology, University of Hawai'i at Mānoa, Honolulu, Hawaii, United States of America; 2 Joint Institute for Marine and Atmospheric Research, University of Hawai'i at Mānoa, Honolulu, Hawaii, United States of America; 3 NOAA Fisheries Service, Pacific Islands Fisheries Science Center, Honolulu, Hawaii, United States of America; 4 Scripps Institution of Oceanography, University of California - San Diego, San Diego, California, United States of America; National Institute of Water & Atmospheric Research, New Zealand

## Abstract

Large-bodied reef fishes represent an economically and ecologically important segment of the coral reef fish assemblage. Many of these individuals supply the bulk of the reproductive output for their population and have a disproportionate effect on their environment (e.g. as apex predators or bioeroding herbivores). Large-bodied reef fishes also tend to be at greatest risk of overfishing, and their loss can result in a myriad of either cascading (direct) or indirect trophic and other effects. While many studies have investigated habitat characteristics affecting populations of small-bodied reef fishes, few have explored the relationship between large-bodied species and their environment. Here, we describe the distribution of the large-bodied reef fishes in the Mariana Archipelago with an emphasis on the environmental factors associated with their distribution. Of the factors considered in this study, a negative association with human population density showed the highest relative influence on the distribution of large-bodied reef fishes; however, depth, water temperature, and distance to deep water also were important. These findings provide new information on the ecology of large-bodied reef fishes can inform discussions concerning essential fish habitat and ecosystem-based management for these species and highlight important knowledge gaps worthy of additional research.

## Introduction

In the terrestrial world, studies of habitat use and resource selection form much of our understanding of wildlife habitat requirements [1], and such studies can potentially provide the basis for effective, spatially based ecosystem management in the marine world as well. Agardy et al. [2] state that marine spatial planning should, at a minimum, include the identification of priority areas as a key element in the design process, and investigations of the relationships between reef fishes and their environment are key in identifying such priority areas. Indeed, many reef fishes have evolved adaptations suiting them to particular reef zones [3], and many studies have described ways in which habitat characteristics affect assemblages of small-bodied reef fishes living in close association with the reef framework and how knowledge of these associations can inform management decisions [4]–[12]. Several authors have also investigated the effects of environmental variables on the distribution of small and large-bodied pelagic [13] and estuarine [14]–[16] fishes. However, less work has focused on the relationships between environmental variables and large-bodied fishes associated with coral reefs [17], especially those in the Mariana Archipelago. Many of these large-bodied fishes are capable of traveling great distances, allowing them to regularly move among various different habitat types. Meyer et al. [18] found that giant trevally (*Caranx ignobilis*) in the Northwestern Hawaiian Islands, made periodic atoll-wide excursions of up to 29 km. These same authors found that green jobfish (*Aprion virescens*) were seasonally attached to core areas of up to 12 km in length. These fish were able to range 19 m across atolls with daily round-trip excursions of up to 24 km [19]. McKibben and Nelson [20] found that grey reef sharks (*Carcharhinus amblyrhynchos*) at Enewetak inhabited home ranges of up to 53 km^2^ and 16 km in length. Finally, Holland et al. [21] found that Bluefin trevally (Caranx melampygus) on patch reefs in Kaneohe Bay, Hawai'i, appear to inhabit fairly stable home ranges for periods of at least a few weeks and possibly up to a year. During the day, tagged fish would patrol back and forth along the face of the patch reef, traveling distances of several hundred meters, and often changing direction at the same point each along the reef. With the exception of most of the pelagic studies and work done on the Great Barrier Reef [22]–[24], most research has focused at relatively small scales—a single estuary, bay, or island—and not at the scale of an entire archipelago. While it is important to investigate small-scale relationships that may differ from island to island and even between different sites within an island, it is no less important to investigate higher-level relationships operating at the archipelagic scale and which may not be apparent from analyses carried out at the site or even island scale [10], [25].

Although tourism is now a key industry, a healthy nearshore coral reef ecosystem able to provide key ecological, economic, and social benefits is no less important [26]. Fish represent the primary natural resource in the Mariana Archipelago, and large-bodied individuals including some species of surgeonfishes (Acanthuridae), jacks (Carangidae), emperors (Lethrinidae), snappers (Lutjanidae), groupers (Serranidae), wrasses (Labridae), and parrotfishes (Scaridae) tend to be preferentially harvested both for local consumption and export [27]–[29]. Large-bodied reef fishes also are of particular importance for a variety of ontogenetic and phylogenetic reasons. Within a species, the largest individuals tend to supply the bulk of the reproductive output for their population [30]. For example, Sudekum et al. [31] found that fecundity in *Caranx melampygus* ranged from approximately 50,000 mature ova for a fish of 760 g (32.8 cm SL) to over 4 million for an individual of 6490 g (64.0 cm SL). Sequential hermaphroditism is common in many teleost fishes including many parrotfishes (e.g. *Scarus rubroviolaceus*) and wrasses (e.g. *Cheilinus undulatus*), with the largest individuals often being disproportionately male or female [32]. Hence, the removal of the largest individuals can have a disproportionate impact on a single gender with associated effects on the reproductive potential of the population. Large-bodied species also tend to have protracted spawning periods [33], and can have a disproportionate impact on their environment, often as apex predators [32], [34] or primary agents of bioerosion [35], [36]. Furthermore, the loss of large-bodied species can have a cascading (direct) or indirect effect on lower trophic levels and ecosystem balance [37]–[39]. Unfortunately, large-bodied individuals tend to be preferentially targeted by fishers, and many large-bodied species are among the most vulnerable to overfishing because they share a suite of life-history characteristics including large size, slow growth rates, and delayed sexual maturity [40]–[42]. Many species of sharks are additionally vulnerable due to their low reproductive rates.

Currently, several fishery targets including snappers, groupers, jacks, surgeonfish, sharks, and large emperors show higher relative abundances in the remote northern islands of the Mariana Archipelago compared to the populated islands of the south, and it has been suggested that abundance and biomass of some taxa has declined in recent decades [28]. Guam, the southernmost major island in the archipelago, has experienced a decline in nearshore reef-associated fish populations accompanied by a sharp decline in catch per unit effort (CPUE) [43]–[45]. Large-bodied species including bumphead parrotfish, humphead wrasse, stingrays, parrotfish, jacks, emperors, and groupers are considered rare in Guam, and it has been suggested that this may be the result of heavy fishing [26]. Technological improvements, which have facilitated the expansion of activities like nighttime SCUBA spearfishing, have resulted in a reappearance of larger species in fishery catch statistics [46]. While it is possible that this resurgence in catches indicates greater targeting of “healthy” populations, it would seem more likely that such increases are related to the use of new technology, representing the next step in the serial depletion cycle seen in many fisheries [47], [48].

At local, regional, and international scales, marine resource management is moving toward a suite of ecosystem-based management approaches including spatial closure and the protection of Essential Fish Habitat [49]. The Western Pacific Regional Fishery Management Council is beginning to integrate ecosystem approaches to management in Guam and the CNMI [26]. However, their current Fishery Ecosystem Plan for the Mariana Archipelago report also states that little is known about the life history, habitat utilization, food habits, or spawning behavior of most of the resident coral reef species. Agardy et al. [2] further argue that true integrated marine protected area planning has yet to be achieved and that the “blind faith” many have placed in often poorly planned and inadequately thought-out marine protected areas carries with it great risk.

Here we test the relative influence of various anthropogenic, physical, oceanographic, and biological environmental factors on the distribution of large-bodied reef fishes in the Mariana Archipelago. Our results show that human populations likely affect the distribution of large-bodied reef fishes but that assemblages are highly variable and other factors including depth, temperature, distance to deep water and many others likely play a role. These results provide additional insight on the relationship between these reef fishes and their environment, can inform discussions of essential fish habitat for these large-bodied reef fishes, and can provide information for managers in their efforts to implement ecosystem-based approaches to fishery management.

## Methods

### Study Area

**Figure 1:**
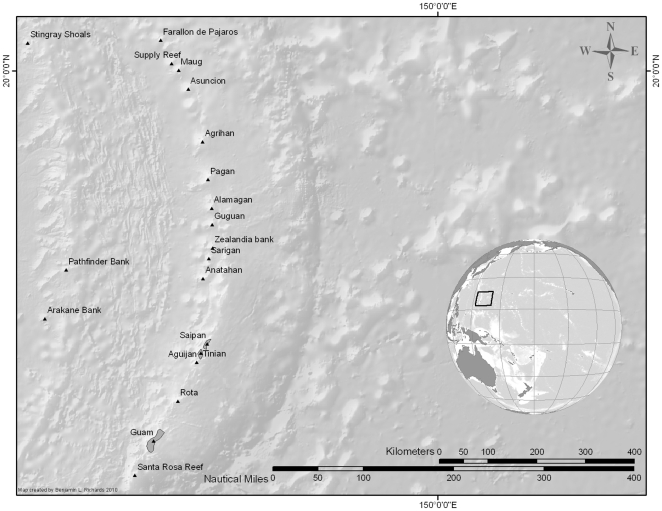
Study area, The Mariana Archipelago showing the location of all islands and banks.

The Mariana Archipelago (politically the CNMI and the US Territory of Guam) is an elongate string of islands stretching 950 km northward in an arc from the island of Guam (13.44°N, 144.76°E) to the Farallon de Pajaros (20.54°N, 144.89°E) (Fig. 1). Islands range in age from 1 to 1.5 million years in the north to >30 million years in the south [50]. The northern islands are volcanically active and topographically complex. The archipelago contains ∼230 km^2^ of shallow-water (<18 m) coral reef habitat, and is the second largest reef area under U.S. jurisdiction in the Pacific [28], [51]. Each island is surrounded by diverse shallow-water coral reefs ranging in size from 0.8 km^2^ around Farallon de Pajaros to 108 km^2^ surrounding Guam [51]. Humans have occupied the Mariana Archipelago since about 3500 B.P. [52], and the present day human population generally declines along a south-to-north gradient from ∼150,000 in Guam and ∼62,000 in Saipan to near zero in the northern islands (U.S. Census Bureau; Census 2000).

### Survey Protocols

Between 2003 and 2009, the Coral Reef Ecosystem Division (CRED) of the NOAA Pacific Islands Fisheries Science Center (PIFSC) conducted 508 large-scale towed-diver surveys for large-bodied (>50 cm Total Length) reef fishes, covering 1038 ha (10.4 km^2^) of the shallow-water (<30 m), coral reef environment around 20 islands and offshore banks in the Mariana Archipelago. These surveys were conducted as part of an ecosystem-wide long-term monitoring program and the lower size threshold of 50 cm TL was chosen to enable observers to concentrate their efforts on the large-bodied, patchily distributed, rare and more mobile species and individuals that were not as effectively sampled by other methods [53]. The 50 cm TL threshold also effectively captured the majority of species often referred to as “apex predators” (e.g., carcharhinids, carangids), as well as the larger herbivores (e.g., scarids) and some species of particular concern (i.e., *C. undulatus*). With the exception of sharks, this size threshold also generates data on the largest of the sexually mature individuals (e.g. Lm for *Caranx melampygus* = 35 cm), which, as mentioned earlier, are expected to have the greatest effect on their environment or population.

Within each year, the entire archipelago was surveyed within a span of 1–2 months. The details of the methodology and its utility for surveying assemblages of large-bodied reef fishes are provided by Richards et al. [53]. In brief, surveys were conducted by means of SCUBA diver-controlled towboards equipped with still and video camera equipment and SeaBird™ SBE39 high-resolution temperature/depth recorders. Divers were towed side-by-side at the end of 60 m lines behind a small survey launch along a depth contour (∼15 m) at a speed of ∼1.5 kt for a duration of 50 minutes per survey. Each survey was partitioned into ten 5-minute survey segments. A global positioning system (GPS) unit mounted on the survey launch recorded survey track points at 5-second intervals, and a custom layback algorithm allowed the diver track to be plotted in a geographic information system (GIS) and merged with spatial data sets (e.g. benthic habitat maps, wave energy estimates). During each survey, one diver recorded, to the lowest possible taxon, all large-bodied reef fishes (>50 cm TL) observed within a 10-m wide lane, focusing observations ahead in a 10-m long moving window. The total length of each fish was estimated to the nearest 5 cm. Cryptic species and fish observed behind the diver were not recorded. Fishes seen leaving and then reentering the survey area were recorded only once. The second diver simultaneously recorded benthic habitat information (e.g. % cover of coral, algae, habitat complexity) as detailed by Kenyon et al. [54]. Habitat complexity is recorded on a six-point scale and is a subjective measure of the topographic diversity or amount of “roughness” of the substrate. Examples of low complexity include sand flat or rubble plains. Areas of steep spur and groove, canyon, pinnacles and walls would be classified as high or very high complexity.

### Data Assimilation

Fish abundance and size data were converted to biomass density (hereafter “biomass”) using the allometric length-weight conversion: W = aTL^b^, where parameters a and b are species-specific constants, TL is total length in mm, and W is weight in grams. Length-weight fitting parameters that most closely matched they survey location and fish size class were obtained from FishBase [55] and Kulbicki et al. [56]. When length-weight fitting parameters used other than total length, length-length conversions were made using formulas from the same sources. A data subset was created that contained species-level biomass values. A relational database and ArcGIS 9.3.1 were used to generate a variety of *in situ* and remotely sensed environmental variables having demonstrated potential to influence the distribution of reef fishes (Table 1). While measures of primary productivity are important to include in ecological models, we have excluded such measures from this analysis for several reasons. Firstly, existing data are unlikely to provide an accurate measure of nearshore primary productivity patterns in this region and at the scale of our study as the standard algorithms are based on the assumption of “optically deep” (i.e. no bottom reflectance) waters. This assumption is violated in the clear, nearshore, shallow-water areas from which the data for this study was derived. Secondly, terrigenous nutrient loading from natural and anthropogenic sources is likely to greatly exceed and mask any open ocean patterns. Hence, remotely sensed primary productivity values derived form open ocean sources would not reflect actual nearshore patterns in primary productivity. Because many of the data layers did not completely overlap with one another or with our towed-diver surveys, it was necessary to further subset the data to create a data matrix that contained only those records with values for all variables.

**Table 1:** Environmental predictor variables included in the boosted regression tree analysis.

Variable Name	Source	Units	Range
Moon phase	calculation from date	days from new	0–29
Time of Day	Index from sequential dive #	#	1–6
Temperature	*in situ* (SBE39)	°C	23.43–30.35
Wave Energy	calculation from NOAA WW3^2^	kW/m	13.76–517.99
Depth	*in situ* (SBE39)	m	1.6–26.5
Distance to 50 m contour	NOAA PIBHMC^3^ bathymetry	m	1–500
Quadrant	calculation	categorical	NE, SE, SW, NW
Human Population/Reef Area	Calculation (2000 US Census)	#/km^2^	0–2445
Complexity	benthic towed-diver visual estimate	index	0–6
Reef Structure	NOAA BHM (Benthic Habitat Maps)^4^	categorical	Reef, Sand, etc
Benthic Cover	NOAA BHM^4^	categorical	Coral, Algae, etc
% Cover – Coral	benthic towed-diver visual estimate	%	0–68.8
% Cover – Macroalgae	benthic towed-diver visual estimate	%	0–87.5
% Cover – Crustose coralline algae	benthic towed-diver visual estimate	%	0–68.75
% Cover – Sand	benthic towed-diver visual estimate	%	8.0–87.5
% Cover – Rubble	benthic towed-diver visual estimate	%	0–56.3
Variety of Cover Types	Calculation from BHM^4^ data	#	1–4
Variety of Structure Types	Calculation from BHM^4^ data	#	1–4

Variables removed due to multi-collinearity		Correlated with:
Mean Sea Surface Temperature	NODC Pathfinder^1^	Human Population/Reef Area
Latitude	*in situ*	Human Population/Reef Area
Longitude	*in situ*	Human Population/Reef Area
Human Population	2000 US Census	Human Population/Reef Area
Reef Area	calculation : 0–30 m hardbottom	Human Population/Reef Area

1 Pathfinder satellite data - http://www.nodc.noaa.gov/SatelliteData/pathfinder4km/.

2 NOAA WaveWatch 3 - http://polar.ncep.noaa.gov/waves/main_int.html.

3 Pacific Islands Benthic Habitat Mapping Center - http://www.soest.hawaii.edu/pibhmc.

4 NOAA Benthic Habitat Maps - http://ccma.nos.noaa.gov/ecosystems/coralreef/us_pac_mapping.html.

### Benthic Habitat

Reef area was calculated based on hard-bottom habitat present within the 0–30 m depth range. Benthic habitat was classified using a combination of *in situ* and remotely sensed data. *In situ* data included % cover of coral, macroalgae, crustose coralline algae (CCA), sand, and rubble and the habitat complexity index we described in the survey protocols. Remotely sensed benthic habitat map layers depicting benthic habitat structure and cover classes, based on IKONOS™ satellite imagery [57] were converted to 5-m resolution raster layers in ArcGIS 9.3.1. Major reef structure (e.g. Rock/Boulder, Pavement, Aggregate Reef) and cover (e.g. Coral, Macroalgae, Uncolonized) categories were defined by NOAA's National Centers for Coastal Ocean Science (NCCOS), using a standard hierarchical classification scheme in which the term benthic cover refers only to biological cover type (i.e. coral, algae) and structure refers to only substrate type, denoting geomorphologic structure (i.e. pavement, sand, reef). The dominant benthic cover and structure were calculated for each survey. The dominant habitat was defined as that assigned to the majority of grid cells within a given survey area. Cover and structure richness values were also calculated based on the “variety” or number of different cover or structure types contained within each survey area.

An attempt was made to incorporate quantitative information on benthic slope and complexity from existing shallow-water multibeam bathymetric maps [58]. However, the level of overlap (∼20%) between the multibeam data (for which the shallowest extent is ∼30 m) and our biological surveys (where the deepest extent is 30 m) was insufficient for analysis. In an effort to nonetheless incorporate information of this type, measures of benthic habitat complexity (as recorded by the benthic towed diver) and distance between each survey and the 50-m isobath were used.

### Oceanography and other Variables

Sea water temperature and diver depth were recorded during each survey using SeaBird™ SBE39 temperature and pressure loggers. Larger-scale sea surface temperature at the island-scale was derived from Pathfinder 4.1 Advanced Very High Resolution Radiometer (AVHRR) Global Climatology [59]. SST values were averaged over a 9 km grid spacing, using nighttime monthly means, averaged over five years of data (2006–2010). NOAA Wave Watch III (WW3) data were used as a proxy for overall wave energy impinging on the shallow reef. WW3 output is a time series (six hour interval) of offshore (deepwater) directional wave energy spectra. Twelve years of WW3 data (1997–2009) were used to calculate average energy flux for each frequency and direction, using deepwater linear wave theory (Equation 1). Direction was binned in 10-degree increments from 0–360 degrees. Period was binned in 2-second increments from 4 seconds to 20 seconds. Frequency is defined as 1/Period yielding a frequency range of 0.05 Hz to 0.25 Hz.

Equation 1. Wave energy flux in watts per meter of wave front.
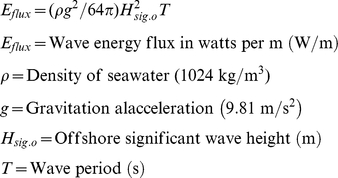



The frequency range with the maximum calculated energy flux was used as an approximation of maximum, and therefore most significant, offshore wave stress. This yielded a maximum offshore wave energy flux (watts per meter of wave front), or maximum power, for each directional bin for each island. The maximal wave energy bin in each quadrant was then used as input for the model. Island quadrant (i.e., NE, SE, SW, NW) was also included as a descriptive variable and was calculated based on the previously calculated directional bins. The time of day for each survey was recorded and moon phase was calculated based on the survey date.

### Anthropogenic Impacts

In the absence of specific data on human impacts such as fishing intensity, human population density per unit reef area was used as a proxy for anthropogenic pressure. Local population estimates came from the 2000 United States Census. Reef area was calculated using a range of GIS layers on bathymetry and bottom type to represent all hardbottom area <30 m deep. Islands were also classified into human population categories of High (>100,000 individuals), Mid (50–1,000 individuals), Low (1–50 individuals), and None. Islands in the Saipan-Tinian-Aguijan complex were each classified as Mid-level based on likely impact level given their close proximity to one another (Tinian is 5 km from Saipan, Aguijan is 8 km from Tinian and 30 km from Saipan), reports that fishers frequently travel among these islands, and information that shore-based fishing has occurred on Aguijan by operations based on the other islands (Trianni, M., pers. comm.). It should be noted that, while we do not have data on the percentage of the population that regularly engages in fishing activities, a large number of immigrant workers reside on Saipan, and these workers do not typically engage in fishing. There are fewer of these workers on Tinian and Rota and therefore, while the total population is lower, the percentage of fishers on these islands is likely greater than on Saipan.

### Data Analysis

The final merged data set (with fish and environmental variables) contained 3711 individual survey segments from 445 surveys conducted around 12 of the 20 islands/banks: Agrihan, Aguijan, Alamagan, Asuncion, Guam, Guguan, Maug, Pagan, Rota, Saipan, Sarigan, and Tinian (Fig. 1). The offshore banks (Stingray Shoals, Pathfinder, and Arakane) and Anatahan were excluded from the analysis, as they were surveyed only during the first year of sampling. Farallon de Pajaros (FDP) was excluded because of the low number of replicates that included all variables. A two-stage analysis was employed using *PRIMER* v6.1.13 [60], followed by Boosted Regression Trees (*BRT*) [61] in the R statistical language v2.6.1 [62]. Data were analyzed at two scales: a larger scale in which data were pooled by island and a smaller scale using data from each towed-diver survey.

*PRIMER* was used for the initial, broad-scale multivariate analysis involving all large-bodied reef fish species at both the island and survey-segment scale to determine the following: 1) similarities in large-bodied reef fish assemblages among islands across the archipelago, 2) which large-bodied reef fish species contributed primarily to any observed patterns and 3) which of the available environmental variables best predict the patterns in the large-bodied reef fish assemblages. The results of the *PRIMER* analyses were further used to help define the scope of the smaller-scale *BRT* analysis (e.g. which large-bodied reef fish species would be analyzed). Overall, *PRIMER* was deemed better able to handle the full multivariate data set, whereas *BRT* was better able to handle the combination of categorical and continuous variables and produced a more detailed description of the relationship between environmental predictor variables and fish response variables.

### Multivariate analyses

Species-level biomass densities were transformed using species-specific dispersion weighting to down-weight highly variable or ‘clumped’ species and to reduce similarity between sampled subregions [63]–[66]. This transformation also down-weighted particularly high biomass species and the need for further transformation was not indicated.

Data were pooled by island to assess large-scale patterns across the archipelago and the relationship among islands. A similarity profile test (*SIMPROF*; [67] was used to determine if significant, interpretable structure in the data existed and, where structure was evident, resemblance matrices were constructed using Bray-Curtis similarity measures [68]. The results of the *SIMPROF* analysis were visualized using hierarchical cluster analysis and nonmetric multidimensional scaling (nMDS) [69]. The *SIMPER* [69] routine was used to determine the relative influence of individual species on the dissimilarity among groups.

### Environmental Correlations

At the smaller scale, Spearman rank correlation was used to identify covarying explanatory variables and, where pairwise rank correlation coefficients were greater than |0.75|, we selected a single variable to represent each variable group. This variable was selected based on data range, interpretability, and ecological theory. Variables estimating % cover were log transformed to reduce skew and all variables were normalized to account for different ranges and measurement scales. The *RELATE* routine [60] was used to test for a relationship between segment-level species biomass and environmental variables and the *BEST* routine [70] was used to select a subset which best explained the overall structure in the large-bodied assemblage.

### Relative Impacts

To measure the relative impact of candidate environmental predictor variables on fish response variables and to assess the specific shape of each relationship, 3711 individual survey segments were modeled simultaneously against 18 continuous and categorical predictor variables (Table 1) using Boosted Regression Trees (*BRT*s) [61]. *BRTs* were constructed using the *gbm* (v1.5-7) [71] and *gbm.step* [61] packages in the *R* statistical language v2.6.1 [62] (Table 2). Regression trees have many desirable properties, including 1) the ability to handle various types of response and predictor variables including both continuous and categorical, 2) the invariance of trees to monotonic transformations of predictors, 3) the ability to model complex interactions in a simple form, and 4) the ability to easily manage missing predictor values with minimal information loss [72]. The two main weaknesses of trees – that they are poor predictors and that large trees can be difficult to interpret – are largely overcome through the use of boosting and consequently, boosted trees are increasingly used in ecological studies [72]–[75].

**Table 2:** Boosted regression tree (BRT) analysis: Optimal parameter settings, predictive performance, and relative influence of environmental variables on total large-bodied reef fish biomass and presence/absence of key species.

Booted Regression Tree Output (optimized)									
Model Parameters	Total Biomass	CAAB	LUBO	MASP	TROB	NAHE	SCRU	CAME	CHUD
**Tree complexity**	3	5	5	1	4	5	4	5	1
**Learning rate**	0.010	0.001	0.010	0.010	0.001	0.01	0.010	0.001	0.010
**Bag fraction**	0.50	0.75	0.75	0.50	0.75	0.75	0.75	0.75	0.75
**# of trees**	1400	5750	1150	2500	3050	3100	1350	3750	1350
**Mean Total Deviance**	0.004	0.289	0.835	0.424	0.390	0.273	0.459	0.335	0.141
**CV Deviance** 1	0.003	0.207	0.595	0.309	0.341	0.228	0.394	0.300	0.131
**SE** 1	0.0001	0.006	0.016	0.008	0.008	0.008	0.007	0.005	0.005
**Percent deviance explained (%)**	25	28	29	27	13	16	14	10	7

Environmental Variables	Relative influence (%)								
Human Population	26.4	19.9	22.6	24.2	30.8	11	7.2	12.7	8.6
Temperature	15.7	17.4	13.5	17.9	12.1	8.1	21.1	18.4	6.8
Depth	11.5	9.8	5.8	6.7	17	14.2	19.2	13.4	49.4
Distance to Deep Water	9.4	12.8	7.8	29.5	6.7	23.6	11.1	13.2	2.8
Wave Energy	6.5	4.1	4.6	1.5	11.9	6.6	11.7	6.9	1.4
Benthic Habitat Structure	4.7	4.6	17.9	6.9	3.3	2.4	3.4	6.1	10.7
Topographic Complexity	4.7	1.7	2.3	7	0.7	1.5	5.8	2.3	1.9
% Macroalgae	4.1	4.6	2.6	0.4	1.6	3	3.1	3.1	2.4
Moon Phase	3.6	5.5	4.1	0.5	5.5	6.7	5	3.2	2.1
Island Quadrant	3	4.6	4.6	0.7	0.9	0.9	2.1	6.1	0.6
Time of Day	2.4	3.9	4.1	0.9	1.4	3.6	2.8	3	2
% Sand	1.8	1.2	2.2	0.1	1.2	2.3	1.5	2.7	0.6
% Coral	1.7	1.9	3.4	2.2	4	4.2	2	3	0.7
Benthic Habitat Cover	1.6	0.5	0.8	0	0.4	1.6	0.9	0.4	4.5
% Crustose Coralline Algae (CCA)	1.5	3.2	2.9	1.2	0.7	5.7	1.7	1.9	0.5
% Rubble	0.8	3.8	0.9	0.4	1.8	3.5	0.8	2.7	0
Variety in Benthic Habitat Cover	0.4	0.2	0.2	0	0.2	1.1	0.4	0.9	0.5
Variety in Benthic Habitat Structure	0.1	0.3	0	0	0	0.1	0	0.1	4.6

Note: Total Biomass values were double-log+1 transformed to achieve pseudo-normality, species were analyzed based on presence/absence. Species selection was based on PRIMER BEST analysis, maximum data density, and management importance. CAAB = *Carcharhinus amblyrhynchos*, LUBO = *Lutjanus bohar*, MASP = *Macolor spp.*, TROB = *Triaenodon obesus*, NAHE = *Naso hexacanthus*, SCRU = *Scarus rubroviolaceus*, CAME = *Caranx melampygus*, CHUD = *Cheilinus undulatus*.

1 Cross-validation (CV) deviance and standard error (SE) is shown as the measure of model performance (the lower the value the better the model performance).

*BRT*s do not assign real probabilities (i.e. *p*-values) and instead use a cross-validation process, which requires some of the data to be held back for model development and validation. Nevertheless, the full data set is still used to fit the model. We used cross validation deviance (CVD) and standard error (SE) as the measure of model performance, where lower values indicate a better model (e.g. a CVD of zero indicates that the model is able to predict new data without any predictive error, and larger CVDs indicate increasing amounts of error). Model optimization was achieved by varying the model parameters: tree complexity, learning rate, and bag-fraction. Tree complexity determines the number of nodes in a tree, while the learning rate is used to shrink the contribution of each tree as it is added to the model. The bag-fraction determines the proportion of the data to be selected at each step in model development and therefore affects stochasticity. We coded a loop routine which sought to minimize model CVD, among models with a minimum of 1000 trees, by adjusting all possible combinations of tree complexity (1, 2, 3, 4, 5), learning rate (0.05, 0.01, 0.001, 0.0001), and bag fraction (0.1, 0.5, 0.75). The combination with the lowest CVD was used to create the final *BRT* model. Relative importance values for each environmental variable were calculated based on the number of times each variable was selected for splitting [72]. Higher relative importance values for a given environmental variable indicate a stronger influence on either total large-bodied reef fish biomass or species distribution. Partial dependency plots were used to interpret the relationship between the environmental predictors and the fish response variables.

Analyses were carried out for total biomass of large-bodied reef fishes and for the occurrence (presence-absence) of eight individual species (Table 3). We were unable to use biomass data at the species level, as assumptions of normality were not valid even after extreme transformation. Species were primarily chosen based on their prevalence in the data, but also because of their relative contribution to overall patterns, and/or their ecological, economic, or management importance (e.g. as apex predators [39], [76], bioeroding herbivores [35], [77], fishery targets, or as IUCN “red list” species) (Table 3). *Macolor niger* and *M. macularis* were grouped as species of *Macolor* because of their ecological similarity and the difficulty distinguishing between the two species in the field. Total biomass values were double (log+1) transformed to achieve normality (or pseudo-normality) [66] and were analyzed based on a Gaussian distribution as required by the technique. Species-level counts were converted to presence-absence by survey segments and were analyzed using a binomial distribution, because the large proportion of zero values precluded normality even after extreme transformation. For each taxanomic group, we also quantified interaction effects among the various predictors (the collinearity and synergistic effect upon predicting the response in question) using the *gbm.interactions* routine [61]. In this routine, the relative strength of interaction fitted by BRT is quantified by the residual variance from a linear model, and the value indicates the relative degree of departure from a purely additive effect, with zero indicating no interaction effects. One can also think of the interaction value as the relative contribution of the interaction between the two predictors towards the overall predictive performance of the individual model (the cv deviance value). For each taxonomic group, we have chosen to report the top two interactions based on interaction value.

**Table 3:** List of taxonomic groups and reasons for inclusion in the analysis.

Taxonomic Group	Data prevalence (# of records)	Contribution to overall pattern	Significance		
			Ecological	Economic	Management
Total Biomass	X (1537)	X	X	X	X
*Carcharhinus amblyrhynchos* (CAAB)	X (122)		X	X	X
*Lutjanus bohar* (LUBO)	X (545)	X			
*Macolor* spp. (MASP)	X (203)	X			
*Triaenodon obesus* (TROB)	X (181)	X	X		X
*Naso hexacanthus* (NAHE)	X (113)		X		
*Scarus rubroviolaceus* (SCRU)	X (226)	X	X	X	X
*Caranx melampygus* (CAME)	X (148)	X	X	X	X
*Cheilinus undulates* (CHUD)	(49)		X		X

Species were chosen based on their prevalence in the data, their relative contribution to overall patterns in the all-species multivariate PRIMER analysis, and/or their ecological, economic, or management importance.

## Results

### General Patterns

**Figure 2:**
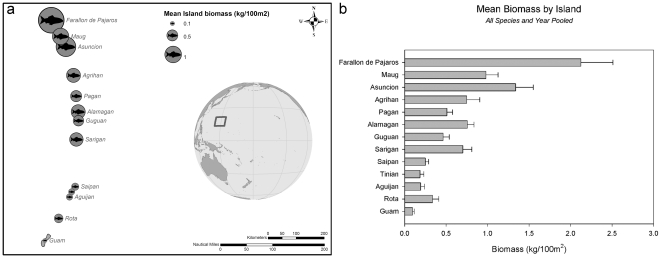
Biomass of large-bodied reef fish (all species and years pooled) in the Mariana Archipelago. Error bars are 1 standard error.

A total of 6280 individual large-bodied reef fish from 67 species were encountered during the survey period. While we were able to reject a null hypothesis of no spatial autocorrelation using Spearman rank correlation between resemblance matrices based on multivariate species-level biomass and the geographic coordinates for each survey segment, the effect size was weak (ρ = 0.113, *p* = 0.001, 999 permutations). The predominant pattern is that of a latitudinal gradient in mean total biomass of large-bodied reef fish (all species and years pooled), ranging from 0.10 g/100 m^2^ (SE 0.02) at Guam to 2.13 g/100 m^2^ (SE 0.39) at Farallon de Pajaros (Fig. 2). The northern (FDP – Sarigan) section of the archipelago has a mean total biomass level nearly twice that of the southern (Saipan – Guam) section (1.81 g/100 m^2^ [SE 0.10] vs. 0.97 g/100 m^2^ [SE 0.05]). This separation between the northern and southern portion of the archipelago was maintained in the restricted data set that incorporates the environmental data.

**Figure 3:**
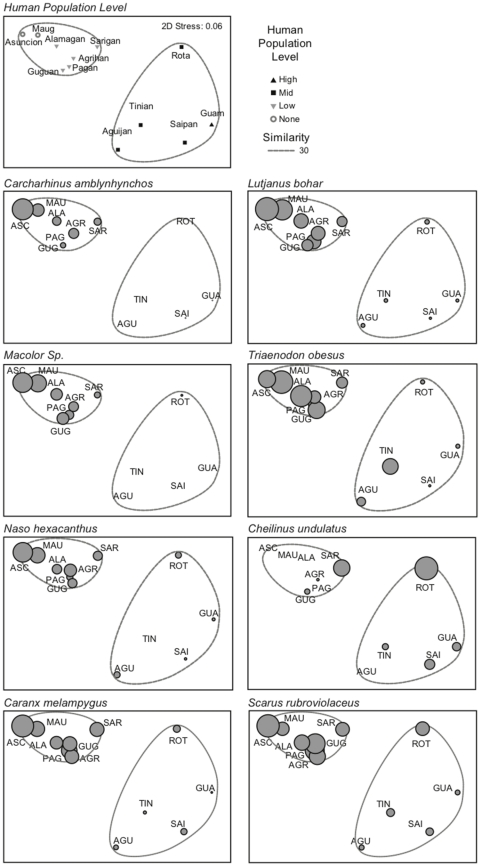
Two-dimensional nMDS ordination plots of islands in the Mariana Archipelago based on multivariate species-level dispersion-weighted biomass of large-bodied (>50 cm TL) reef fishes and Bray-Curtis similarities. Cluster contours represent significant SIMPROF groups (∼30% similarity within groups). In the first panel, symbols reflect level of human population. Aguijan is categorized as “Mid” human population due to the influence of nearby Tinian and Saipan as well as reported visitation by fishers from Guam. Subsequent panels are the same nMDS as panel 1, with symbols representing relative biomass of each species.

This pattern was evident in the hierarchical *CLUSTER* and n*MDS* analysis, based on species-level biomass density, and the *SIMPROF* test, which showed a distinct separation between the northern islands, which showed absent (None) to Low human population levels, and those in the south, which showed Mid to High populations (Global R = 0.044, *p* = 0.001) (Fig. 3). This dissimilarity was driven primarily by five major taxa (*Carcharhinus amblyrhynchos*, *Lutjanus bohar*, species of *Macolor*, *Triaenodon obesus*, and *Naso hexacanthus*), all of which showed higher biomass values at the northern, less populated islands (Table 4). *Carcharhinus amblyrhynchos* and species of *Macolor* were observed only in the northern islands, with the highest biomass densities observed at Asuncion Island (0.57 g/100 m^2^ [SE 0.16] and 0.21 g/100 m^2^ [SE 0.08], respectively). Although *Lutjanus bohar* and *Naso hexacanthus* were observed at both the northern and southern islands, biomass densities were higher at the northern islands, with the highest levels of both observed also at Asuncion (0.28 g/100 m^2^ [SE 0.04] and 0.11 g/100 m^2^ [SE 0.03], respectively). *Triaenodon obesus* were observed at both the northern and southern islands; however, biomass densities were higher in the northern islands with the highest levels at Maug and Alamagan Islands (0.14 g/100 m^2^ [SE 0.02] and 0.12 g/100 m^2^ [SE 0.03], respectively).

**Table 4:** *SIMPER* analysis results showing species contribution to the dissimilarity between significant *SIMPROF* groupings based on species-level biomass (dispersion-weighted) and human population.

	Average Biomass (disp)					
Species	(+) Humans	(−) Humans	Average Dissimilarity	SD	% Contribution	Cummulitave % contribution
*Carcharhinus amblyrhynchos*	0.04	0.21	9.75	1.84	18.74	18.74
*Lutjanus bohar*	0.09	0.24	9.27	4.72	17.81	36.55
*Macolor* spp.	0.05	0.18	7.61	4.66	14.62	51.17
*Triaenodon obesus*	0.06	0.11	3.67	1.32	7.05	58.22
*Naso hexacanthus*	0.03	0.09	3.56	3.33	6.84	65.06

High-Mid human population group is designated as (+) Humans while Low-None group is designated as (−) Humans.

### Fish-Habitat Relationships

**Figure 4:**
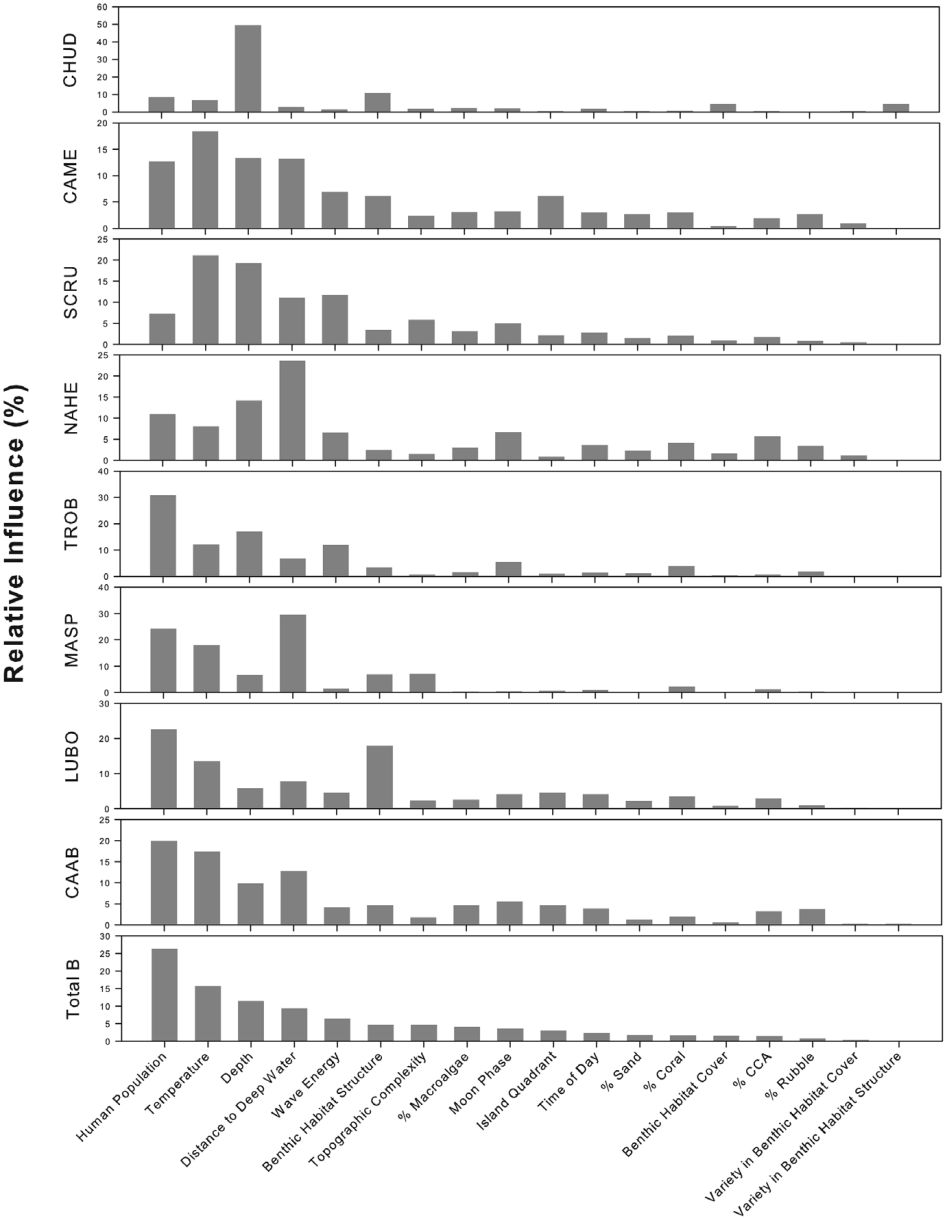
Relative importance plots for boosted regression tree analysis of total large-bodied reef fish biomass (all species pooled) and presence/absence of key species. Species selection was based on maximum data density, and management and ecological importance. CAAB = *Carcharhinus amblyrhynchos*, LUBO = *Lutjanus bohar*, MASP = *Macolor* spp., TROB = *Triaenodon obesus*, NAHE = *Naso hexacanthus*, SCRU = *Scarus rubroviolaceus*, CAME = *Caranx melampygus*, CHUD = *Cheilinus undulatus*.

**Figure 5:**
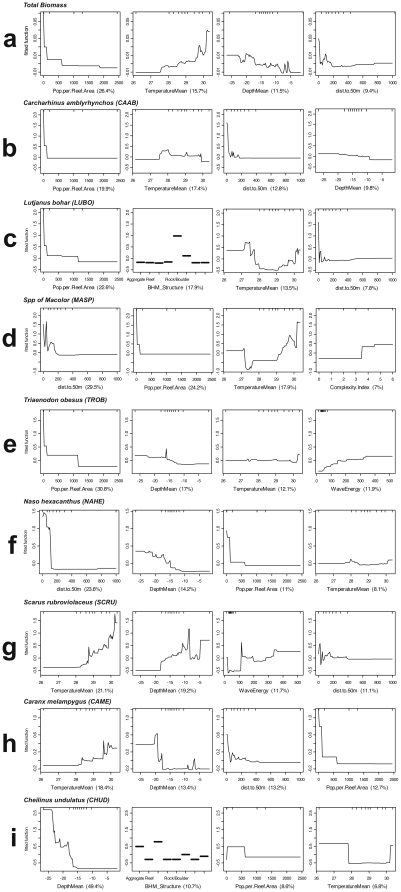
Partial dependency plots for boosted regression tree analysis of total large-bodied reef fish biomass (all species pooled) and presence/absence of key species. Species selection was based on maximum data density, and management and ecological importance. CAAB = *Carcharhinus amblyrhynchos*, LUBO = *Lutjanus bohar*, MASP = *Macolor* spp., TROB = *Triaenodon obesus*, NAHE = *Naso hexacanthus*, SCRU = *Scarus rubroviolaceus*, CAME = *Caranx melampygus*, CHUD = *Cheilinus undulatus*.

The null hypothesis of no relationship between large-bodied reef fish species distribution and the predictor variables was rejected (ρ = 0.109, *p* = 0.001). Of the 15 continuous predictors, human population density (human population per reef area) provided the *BEST* match (corr. = 0.197). Excluding human population density from the analysis resulted in a *BEST* match that, while having a lower correlation (0.106), indicated four environmental variables as significant predictors (Moon phase, % Coral Cover, % Sand Cover, and Variety in Habitat Structure). We were able to quantify the relative influence of each of the 18 continuous and categorical environmental variables using Boosted Regression Trees (Fig. 4 & Table 2) and were able to interpret the specific nature of each relationship in more detail using the resulting partial dependency plots (Fig. 5).

### Total large-bodied reef fish

**Figure 6:**
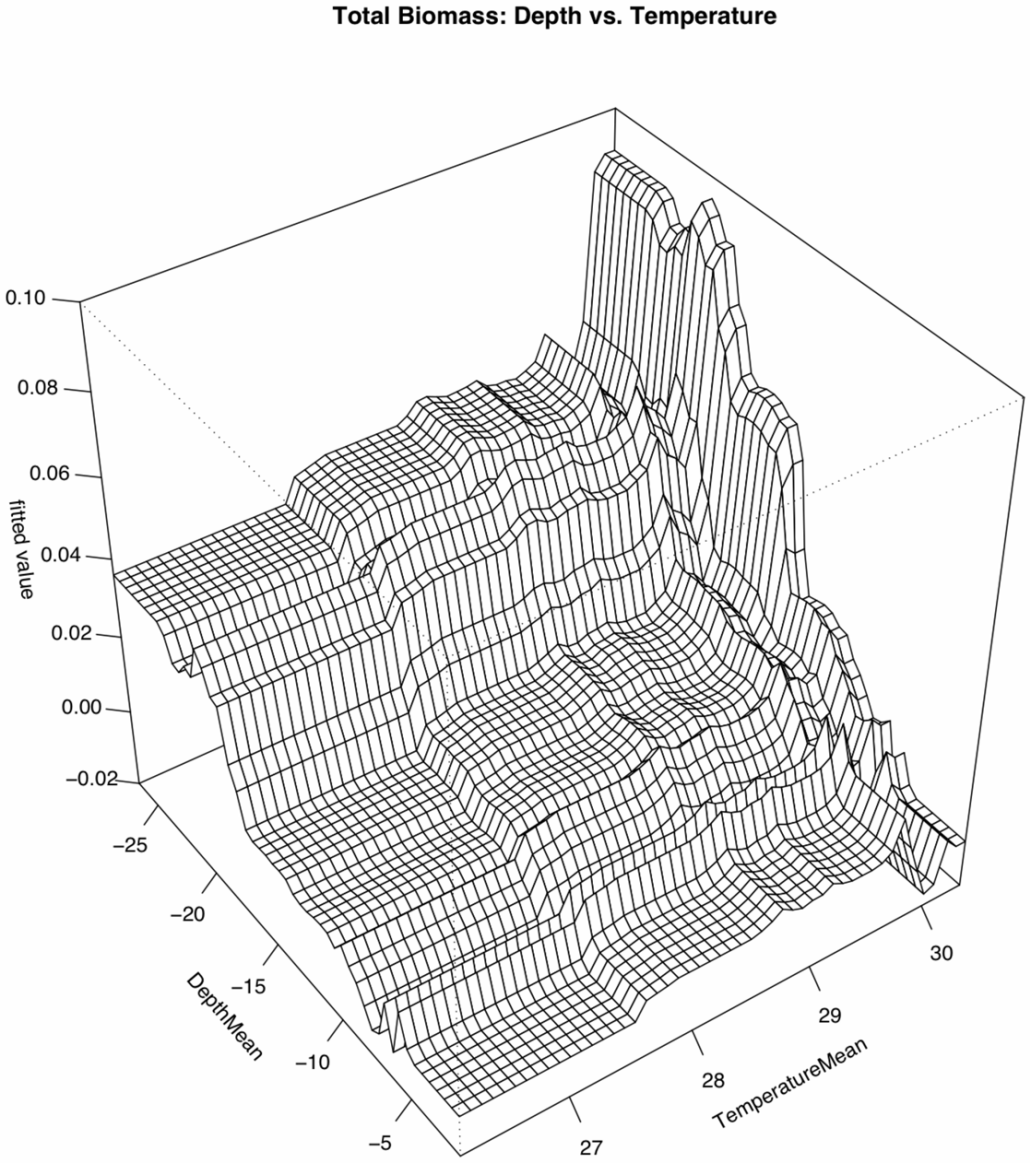
Pairwise interaction between depth and temperature with respect to total biomass of large-bodied reef fish. The effect of temperature on total large-bodied reef fish biomass is magnified in areas of warm water and the effect of temperature is magnified at deeper depths.

Three relationships contributed most strongly to predicting total large-bodied reef fish biomass (Figs. 4 & 5a). Biomass was highest in areas with low human population density (relative influence [RI] = 26.4%) and decreased as human population increased. Biomass also gradually increased as *in situ* water temperature increased (RI = 15.7%), rose steeply once water temperature neared 30°C, and peaked at just over 30°C. Total biomass density of large-bodied reef fish also increased with increasing depth (RI = 11.5%) and with proximity to deep water (RI = 9.4%). Overall model CVD was 0.003 with a second order interaction between depth and temperature (Table 5). The effect of depth was exacerbated in the warmest areas (near 30°C) and the effect of temperature was similarly exacerbated in the deepest areas (deeper than ∼20 m). In general biomass is highest at deeper depths (>20 m) and at higher temps (above 30°) (Fig. 6). However, biomass is also higher below 20 m across the entire temperature range.

**Table 5:** Pairwise interactions between predictor variables used to relate total biomass and occurrence of each taxa to environment.

Taxa	Predictor	Predictor	Interaction value	Interaction Summary
Total Biomass	Depth	Temperature	0.03	Deeper Depth+Higher Temp = Higher Biomass
CAAB	(BHM_Structure)	Temperature	32.31	
	Depth	Human Population	28.25	Depth >15 m+0 people = More CAAB
CAME	% CCA	Depth	11.78	Depth >20 m+CCA >20% = More CAME
	(BHM_Structure)	Depth	7.64	
CHUD	*NA*	*NA*	*NA*	*To few samples to model interactions*
LUBO	Wave Energy	Temperature	25.12	Temp <27 or >29+Higher Wave Energy = More LUBO
	Depth	(Island Quardrant)	9.17	
MASP	*NA*	*NA*	*NA*	*To few samples to model interactions*
NAHE	Distance to Deep Water	(BHM_Structure)	20.82	
	Distance to Deep Water	(BHM_Cover)	6.62	
SCRU	Wave Energy	Depth	27.61	Shallow depth+low to moderate Wave Energy = More SCRU
	% Sand	Wave Energy	11.92	% Sand <20+Wave Energy between 100 & 200 = More SCRU
TROB	Depth	Human Population	20.99	Depth >15 m+0 people = More TROB
	Wave Energy	Human Population	4.17	

*Interactions displayed are the top two for each taxonomic group (based on value) that involved the 8 predictors offering the highest contribution to the model displayed in* *Figure 5* *. Only one interaction is displayed for Total Biomass as none of the lower interactions had values greater than 0.01. Interaction value indicates the relative degree of departure from a purely additive effect; with a value of zero indicating that no interaction is present. A summary description is given for the association of the peak in each of 9 taxonomic groups and the pairwise interactions for those predictor variables showing a clear relationship (for example positive, negative, or modal) with the taxa in* *Figure 5*.

*Categorical variables are noted with “()”.*

*NA indicates sample sizes insufficient to model interactions.*

### *Carcharhinus amblyrhynchos*

**Figure 7:**
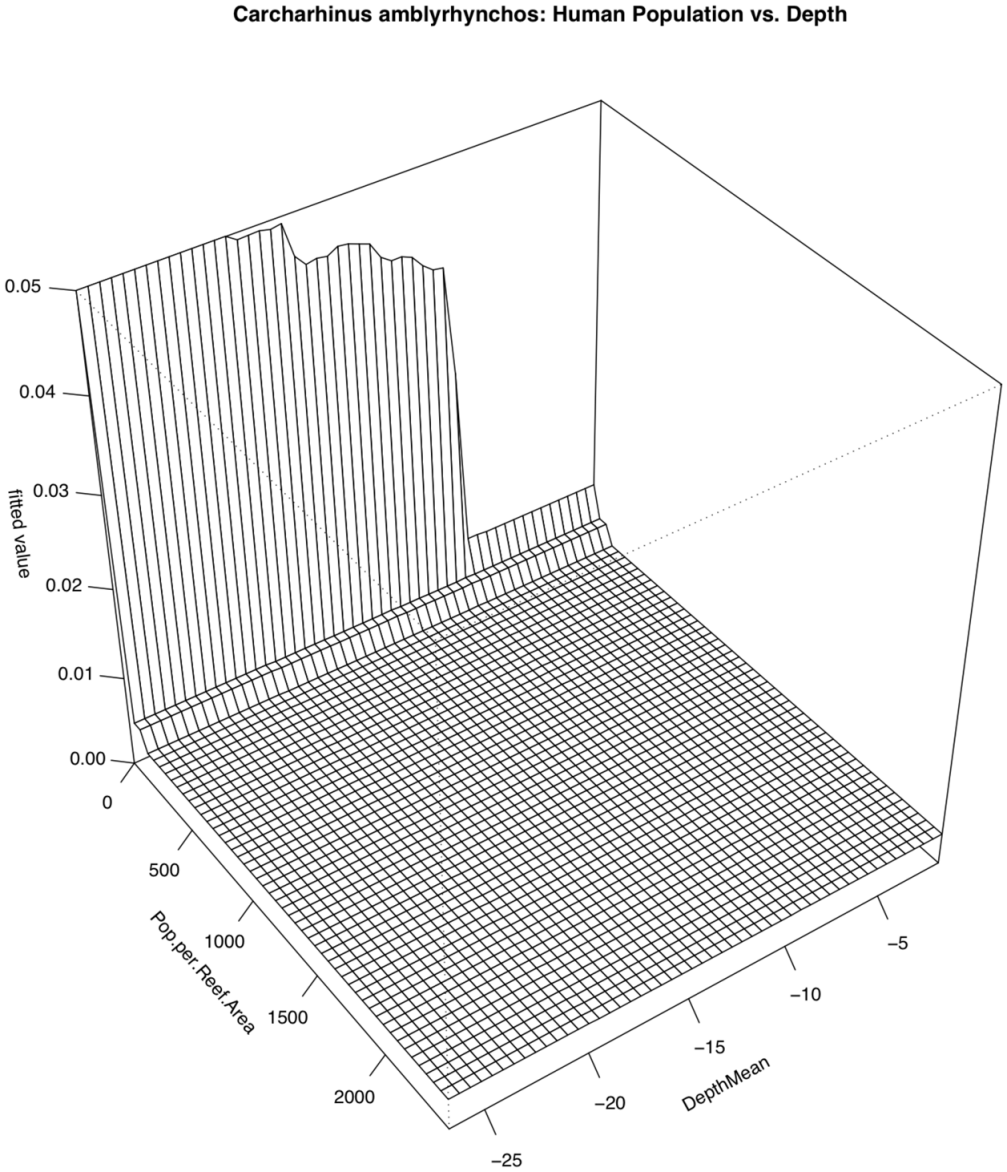
Pairwise interaction between human population density and depth with respect to occurrence of *Carcharhinus amblyrhynchos*. Depth is only influential in areas of low human population density and the effect of human population density is reduced in shallow waters.

Three relationships contributed most strongly to predicting the occurrence (presence/absence) of *Carcharhinus amblyrhynchos* (Figs. 4 & 5b). These were a steep negative relationship with human population density (RI = 19.9%), a somewhat variable relationship with *in situ* water temperature (RI = 17.4%), and a negative relationship with distance to deep water (RI = 12.8%). Sightings were most frequent where water temperatures were just below 28°C and decreased gradually with a smaller peak at 29°C. Overall model CVD was 0.207 with second order interactions between habitat structure and temperature as well as between depth and human population (Table 5). While the partial dependency plots show that both human population density and depth are correlated with distribution of *C. amblyrhynchos* (with higher occurrences in areas of low human population density and in deep water), the correlation with depth is partially dependent on local human population density. The effect of depth is weaker (or almost non-existent) where human population density is high and stronger (or only seems to matter) where there are fewer (to zero) people (Fig. 7). Similarly, the effect of local human population density is much reduced in shallower depths.

### *Lutjanus bohar*

**Figure 8:**
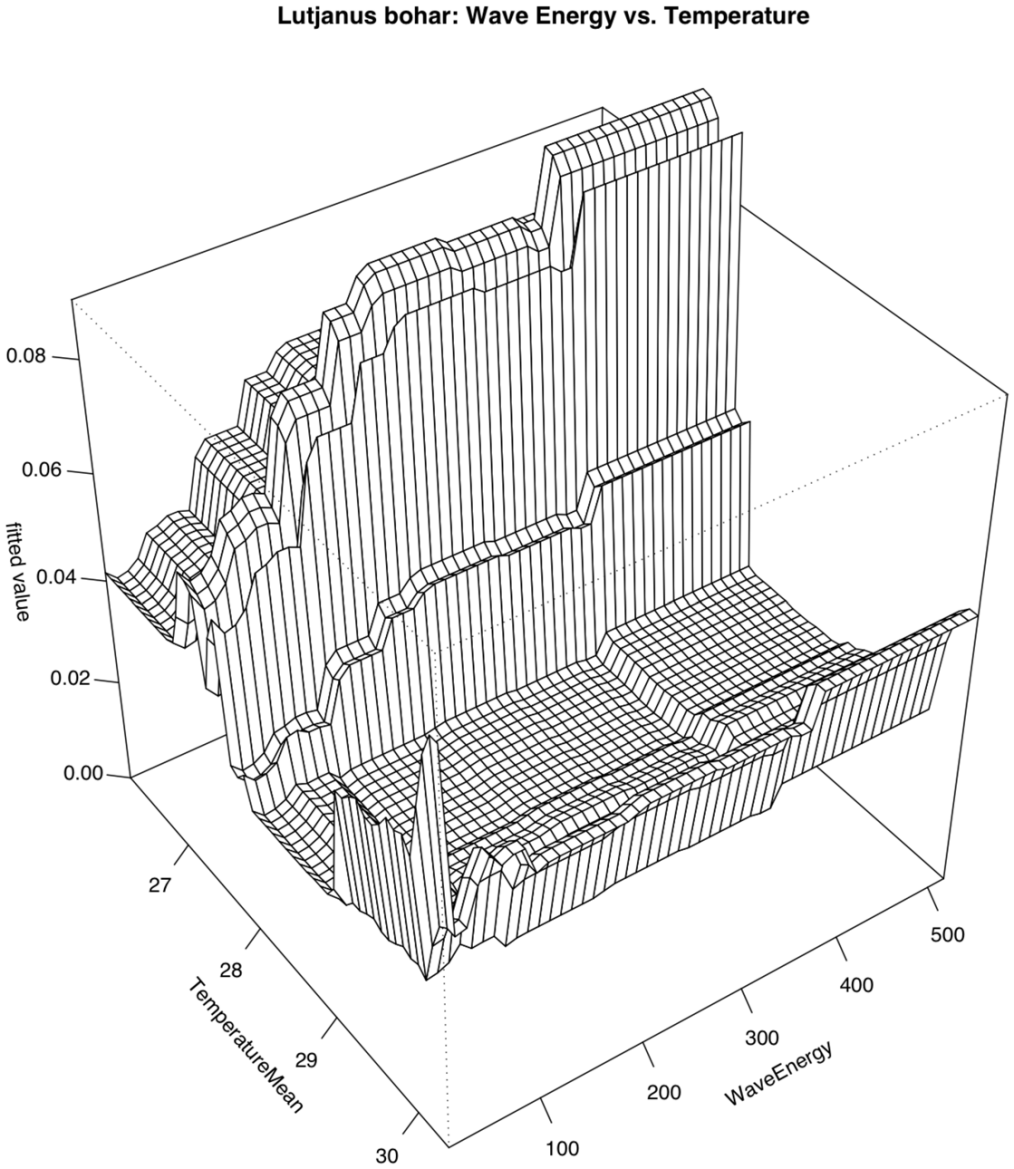
Pairwise interaction between wave energy and temperature with respect to occurrence of *Lutjanus bohar*. The effect of temperature is magnified by wave energy while wave energy is primarily influential at temperatures below 28°C.

Two relationships contributed most strongly to predicting the occurrence of *Lutjanus bohar* (Figs. 4 & 5c). A negative relationship with human population density was the strongest predictor (RI = 22.6%). This was followed by a relationship with benthic habitat structure (RI = 17.9%) for which *Lutjanus bohar* sightings were highest in areas classified as “Rock/Boulder”. Sightings decreased as water temperatures warmed from 27°C to 28°C and increased again once water temperatures reached 29°C (RI = 13.5%). Sightings also dropped steeply as distance to deep water increased (RI = 7.8%). Overall model CVD was 0.595 with second order interactions between wave energy and temperature as well as between depth and island quadrant (Table 5). *L. bohar* occurred most frequently in low temperature (<27.5°C) high wave energy waters (>250 kW/m). The effect of wave energy was stronger at these lower water temperatures and temperature appeared to have more of an effect at higher wave energies (Fig. 8). The interaction between depth and island quadrant suggests that the effect of depth was not universal across island quadrants, but this type of interaction (between categorical and continuous variables) is difficult to interpret.

### *Macolor* spp

Three relationships contributed most strongly to predicting the occurrence of species of *Macolor* (Figs. 4 & 5d). An overall negative relationship with distance to deep water was the strongest predictor (RI = 29.5%). This was followed by a steep negative relationship with human population density (RI = 24.2%). Sightings of species of *Macolor* also decreased as water temperatures warmed from 27°C to 29°C and increased again once water temperatures exceeded 29°C (RI = 17.9%). Sightings of species of *Macolor* also increased with increases in observed benthic habitat complexity (RI = 7%). Overall model CVD was 0.309 and sample sizes were insufficient to model secondary interactions.

### Triaenodon obesus

**Figure 9:**
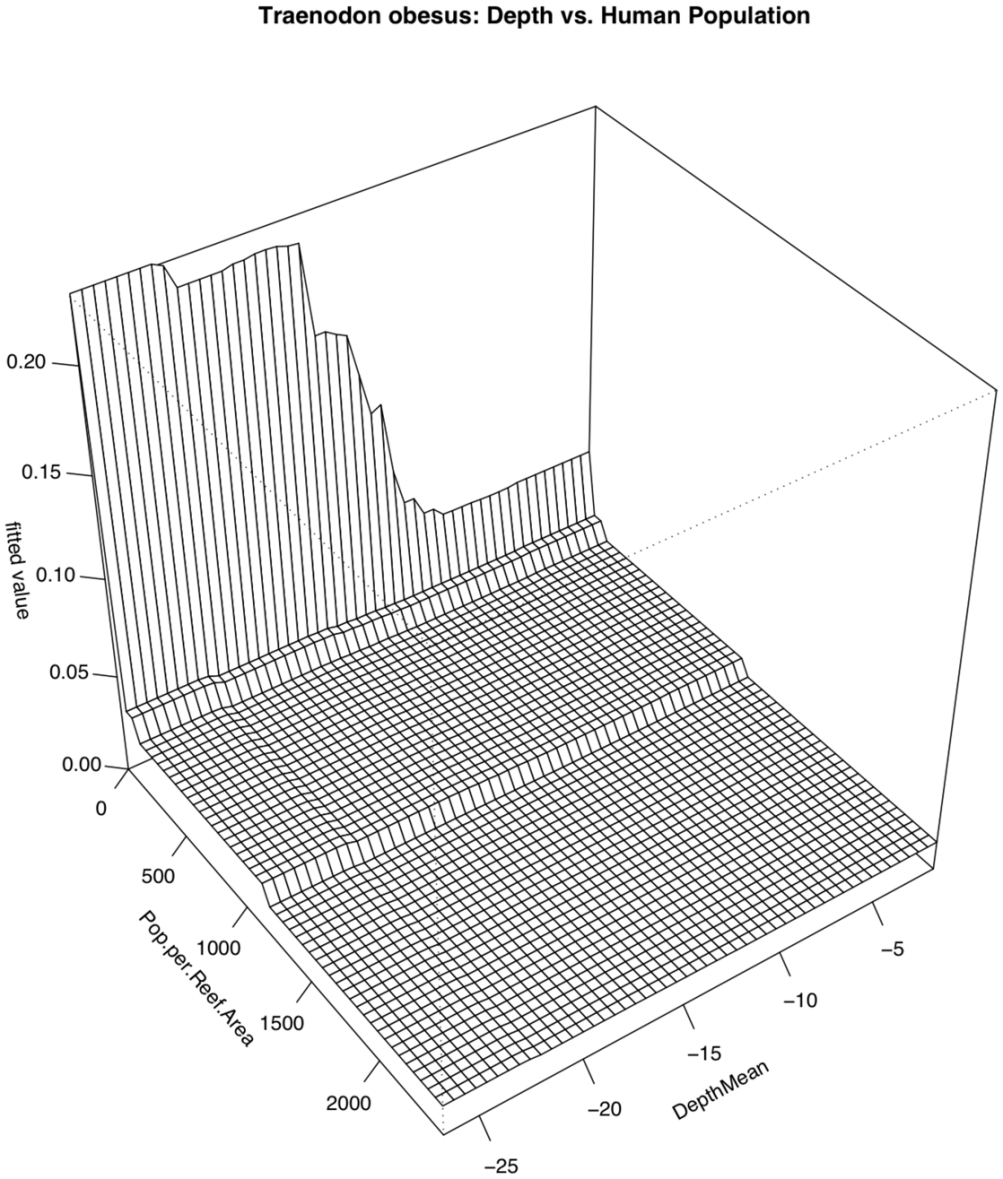
Pairwise interaction between human population density and depth with respect to occurrence of *Triaenodon obesus*. The effect of depth is influential only at the lowest human population densities and the effect of human population density is reduced in shallow areas.

**Figure 10:**
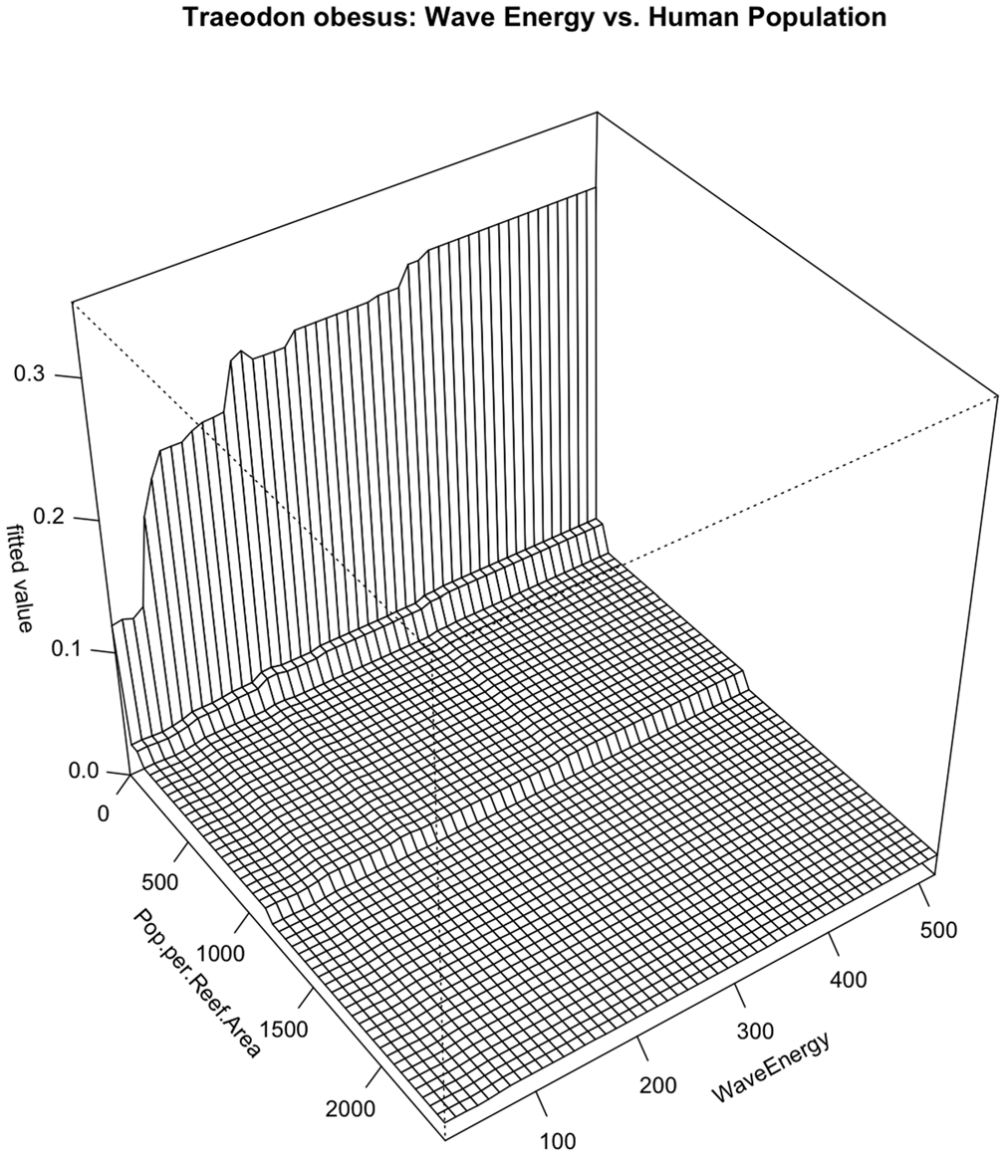
Pairwise interaction between human population density and wave energy with respect to occurrence of *Triaenodon obesus*. The effect of wave energy is influential only at the lowest human population densities and the effect of human population density is magnified in higher wave energy areas.

A negative relationship with human population density was the strongest predictor of sightings of *Triaenodon obesus* (RI = 30.8%) with a steep decline in presence between 0 and approximately 200 humans/km^2^ of reef and another drop once human population density reached 1000 individuals/km^2^ (Figs. 4 & 5e). A slightly positive relationship existed between sightings of *Triaenodon obesus* and depth (RI = 17%), with a spike in sightings at depths between 15 and 20 m. Slightly positive relationships also existed between sightings of this species and temperature (RI = 12.1%) and wave energy (RI = 11.9%). Overall model CVD was 0.341 with second order interactions between human population density and depth and wave energy (Table 5). In a pattern reminiscent of that seen in *C. amblyrhynchos*, the relationship between *T. obesus* and depth is partially dependent on local human population density. The effect of depth is weaker (or almost non-existent) where human population density is high and stronger (or only seems to matter) where there are fewer (to zero) people (Fig. 9). Similarly, the effect of local human population density is much reduced in shallower depths. Similarly, wave energy was only influential when human population density was low and the effect of human population density appeared to be reduced in areas with low wave energy (Fig. 10).

### *Naso hexacanthus*

A steep negative relationship with distance to deep water most strongly predicted sightings of *Naso hexacanthus* (RI = 23.6%), with fitted values dropping to near zero as distances to the 50 m isobath exceeded 200 m (Figs. 4 & 5f). A positive relationship also existed with depth (RI = 14.2%), and there was a steep negative relationship with human population density (RI = 11%). Overall model CVD was 0.228 with second order interactions between distance to deep water and benthic structure and cover (Table 5). The interaction between distance to deep water and benthic structure and cover (both categorical variables) suggests that the effect of distance to deep water was stronger in certain structure or cover classes as opposed to others, and that the relationship with cover and structure is partially dependent on proximity to deep water. Again however, this type of interaction (between categorical and continuous variables) is difficult to interpret.

### *Scarus rubroviolaceus*

**Figure 11:**
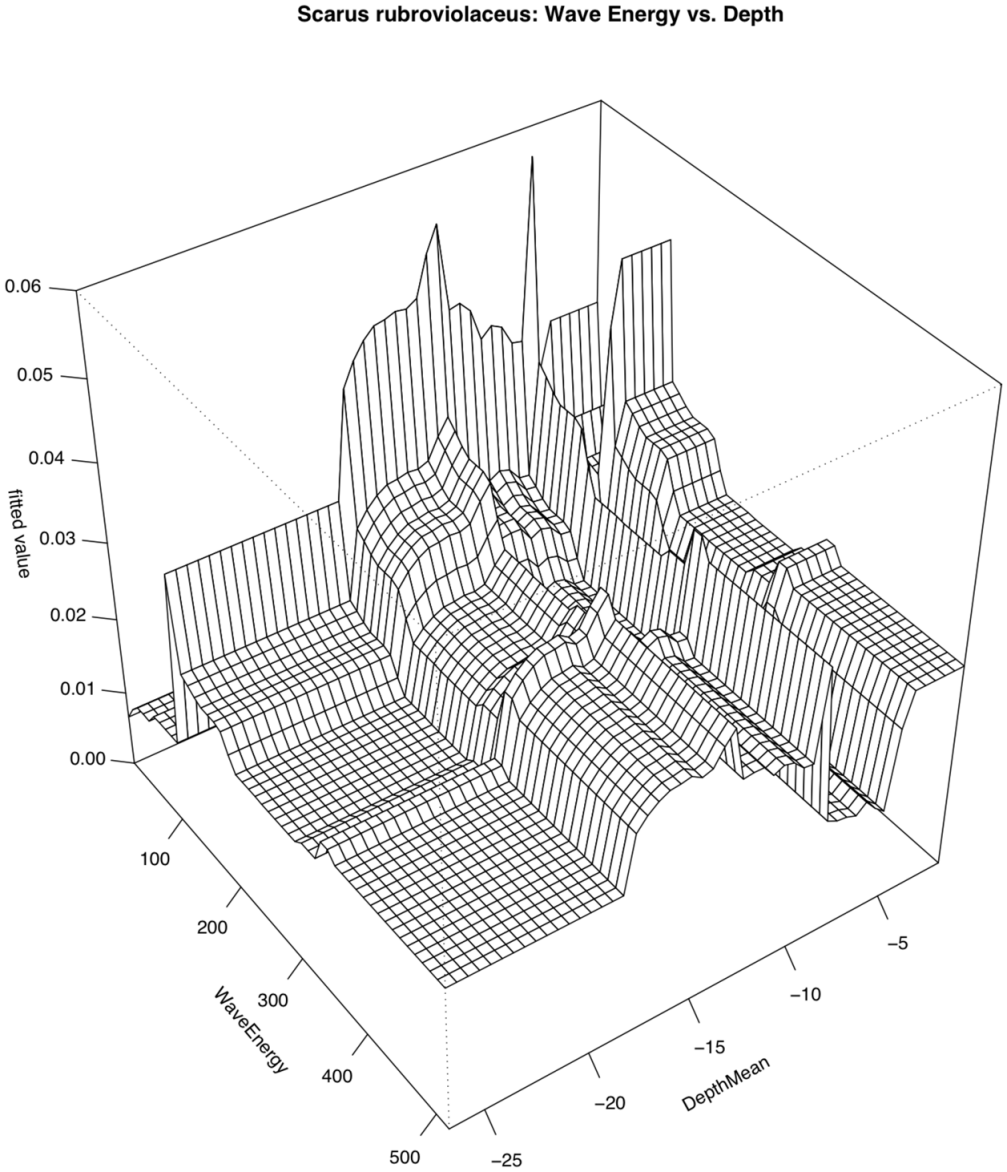
Pairwise interaction between depth and wave energy with respect to occurrence of *Scarus rubroviolaceus*. The effect of wave energy is influential only in depths shallower than 15 m. The effect of depth is somewhat magnified in lower wave energy areas.

**Figure 12:**
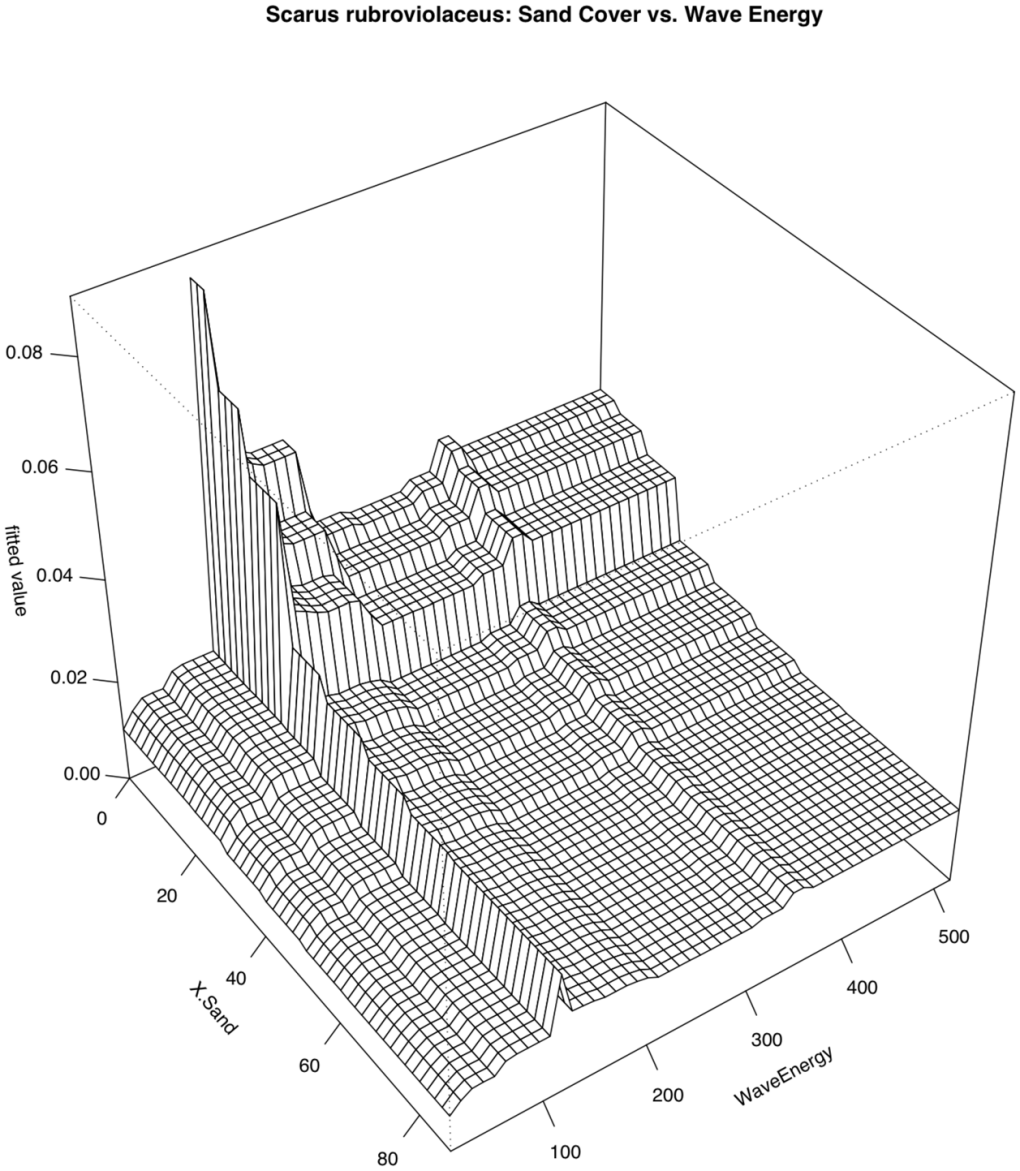
Pairwise interaction between wave energy and sand cover with respect to occurrence of *Scarus rubroviolaceus*. The effect of wave energy was highest in areas with low sand cover and the effect of sand cover was greatest in areas were wave energy was between 100 and 200 kW/m.

Relationships with temperature and depth contributed most strongly to predicting the occurrence of *Scarus rubroviolaceus* (RI = 21.1% and 19.2%, respectively) (Figs. 4 & 5g The relationship with temperature was generally positive but was highly variable. The relationship with depth was also highly variable but sighting frequency was highest in depths near 10 m or less than 5 m. Highly variable relationships also existed with wave energy (RI = 11.7%) and distance to deep water (RI = 11.1%). There was not a strong relationship between the occurrence of this species and local human population density. Overall model CVD was 0.394 with second order interactions between wave energy and depth and sand cover (Table 5). The interaction between depth and wave energy is complicated. The relationship between *S. rubroviolaceus* and depth appears to be most pronounced in low wave energy environments (Fig. 11). As one might expect, the relationship between *S. rubroviolaceus* and wave energy is apparent only at depths shallower than 15 m and is strongest at depths shallower than 5 m. The effect of wave energy is also much reduced in areas of high sand cover. The effect of sand is highest at wave energies of between 100 and 200 kW/m (Fig. 12).

### *Caranx melampygus*

**Figure 13:**
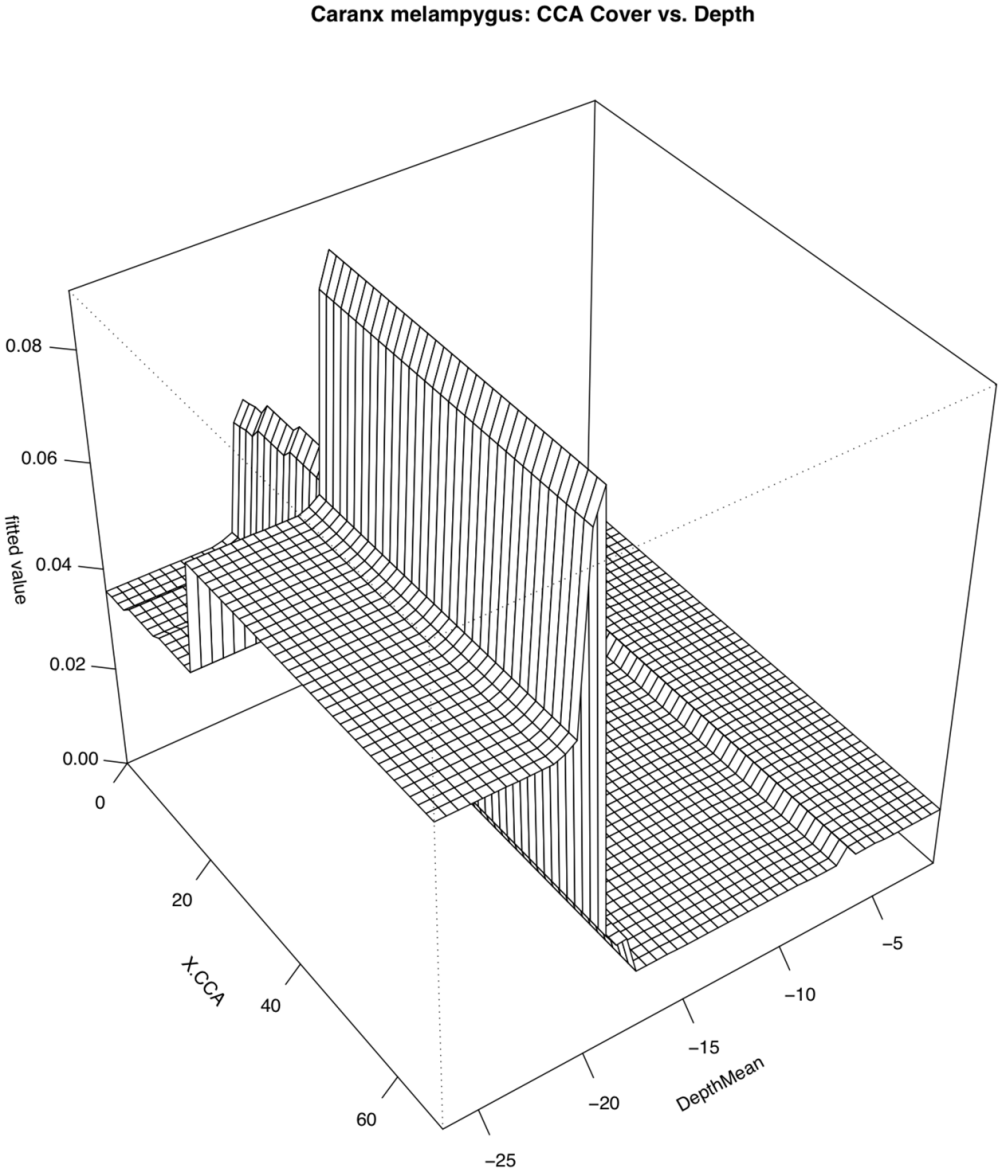
Pairwise interaction between depth and percent cover of crustose coralline algae (CCA) with respect to occurrence of *Caranx melampygus*. The effect of CCA was greatest at depths greater than 15 m while the effect of depth was reduced in areas with lower CCA cover.

Temperature, depth, distance to deep water, and human population density all contributed to predicting the occurrence of *Caranx melampygus* (RI = 18.4%, 13.4%, 13.2%, 12.7%, respectively; Figs. 4 & 5h). The relationship with temperature and depth were positive but highly variable, while the relationship with distance to deep water was negative. Sighting frequency declined steeply as human population density increased from zero. Overall model CVD was 0.300 with second order interactions between depth and percent cover of crustose coralline algae (CCA) and benthic structure (Table 5). The effect of percent cover of CCA was almost non-existent and depth shallower than 17 m and the effect of depth seems to have been reduced in areas with low CCA cover (Fig. 13). The interaction between depth and benthic structure (another categorical variable) suggests that the effect of depth was stronger in certain structure classes as opposed to others, and that the relationship with structure is partially dependent on depth.

### *Cheilinus undulatus*

Depth most strongly contributed to predicting the occurrence of *Cheilinus undulatus* with a greater number of sightings at depths greater than 15 m (RI = 49.4%) (Figs. 4 & 5i). Sightings were also higher in areas classified as pavement and aggregate reef (RI = 10.7%). Human population density had a relative influence of 8.6%, with higher occurrences of *Cheilinus undulatus* in areas where human population density was between 0 and 750 individuals per km^2^ of reef. Overall model CVD was 0.131 and sample sizes were insufficient to model secondary interactions.

## Discussion

The islands of the Mariana Archipelago span a wide range of geographic, environmental, and anthropogenic gradients. The archipelago spans nearly ten degrees of latitude and ranges from relatively large, heavily populated, carbonate islands in the south to small, remote, unpopulated, volcanic islands in the north. While one might expect significant spatial structure in this type of data, a test for spatial autocorrelation indicates that our data are highly variable across space and that any effect of spatial autocorrelation is weak and likely overshadowed by the environmental variables used out model. Nevertheless, a number of the environmental and anthropogenic variables selected for analysis covaried, thus limiting our ability to draw definitive conclusions about the effect of any one variable to the exclusion of others. For example, the north-south gradient in human population density followed similar gradients in latitude, mean sea surface temperature, open ocean primary productivity and island size. Consequently, while we feel that human populations density is the most likely causative factor, and a substantial body of literature exists that would support such a conclusion [32], [38], [78]–[84], we are nevertheless, not able to say with certainty that human population density is the causative factor to the exclusion of all others and our results should be used to indicate factors that appear to be important in shaping the spatial distribution of large-bodied reef fishes in this geographic area.

Measurements of *in situ* temperature factored highly in our analysis of relative influence and many studies have focused on the importance of temperature in the ontogeny of fishes as well as in structuring marine and aquatic communities. Francis [85] discusses the importance of sea surface temperature in determining the year class strength of New Zealand snapper while Worm et al. [86] show that patterns of diversity in top predators can be correlated with thermal fronts and patterns in the distribution of dissolved oxygen. In their 1980 study of habitat preference and fisheries oceanography, Magnuson et al. [87] showed that sea surface temperature was the best predictor of catch per unit effort (CPUE). These authors do, however, suggest that, as with any environmental correlation, the causal hypothesis regarding temperature needs laboratory verification as the finding that fish distributions following temperature gradients does not necessarily mean that temperature itself is the forcing function. Rather, it is possible that higher trophic-level fishes may be responding to the availability of prey species that are the ones responding to temperature. Furthermore, it is clear that fish assemblages are not responding directly to human population density, but are rather responding to a variety of direct and indirect factors (e.g. fishing, sedimentation, habitat degradation, etc.) related to human population density. It should also be noted that the coral reef system is enormously complex, with myriad interacting factors affecting species distributions through a variety of direct and indirect ways. Furthermore, the assemblages of large-bodied reef fishes we describe are rare, highly mobile, and patchily distributed. Hence, even our most complete model leaves the majority of deviance unexplained. That being said, the key findings of our study, such as the high relative importance of human population density and the effect of depth and distance to deep water remain important considerations for management of the marine resources of Guam and the Commonwealth of the Northern Mariana Islands and are consistent with research on other taxa from other locations [32], [34], [80], [81], [88]. While the low relative influence of small-scale environmental factors (i.e. benthic cover and habitat heterogeneity) that have been previously identified as important in the distribution of small-bodied reef fishes [10], [89]–[91] is intuitive given their life history, to our knowledge this has seldom been demonstrated in the literature regarding this area. It stands to reason that small-scale factors are of prime importance to those species that interact most directly with the reef either for food or shelter [92]. Chabanet et al. [90] found no relationship between habitat cover and the abundance of planktivores or carnivores at Reunion Island, and suggest that the relationship between the abundance of fish and coverage of living coral may be stronger in shallow water where fish remain in closer physical proximity to the substrate. However, as Levins [93] suggests, the perceived “grain size” or “resolution” of habitat depends on the body size of the individual animal. Hence, for wide-ranging large-bodied species small-scale differences in benthic cover are likely less important.

### Anthropogenic Impacts

Community structure, relative biomass, and species occurrence differed substantially between the heavily populated southern islands and the more remote and unpopulated islands in the north. Overall biomass of large-bodied reef fish in the northern islands was nearly twice that found in the southern islands. Several taxa such as *Carcharhinus amblyrhynchos* and species of *Macolor* were abundant in the northern islands and nearly absent in the southern islands (Fig. 3). Other species such as *Lutjanus bohar*, *Triaenodon obesus*, *Scarus rubroviolaceus*, and *Caranx melampygus* were found both north and south, but biomass densities were much lower at the southern islands. At present, we do not know of a robust direct measure of fishing pressure in the Mariana Archipelago, so the evident relationship between many of the taxa in our study and local human population density cannot be directly attributed to the effects of fishing. While anthropogenic habitat destruction cannot be ruled out as a possible cause, we would expect anthropogenic habitat destruction to manifest itself through changes in biological benthic cover (e.g. shifts from coral to algae) and possibly changes in small-scale substrate complexity (e.g. sedimentation, coral death and erosion). As these habitat factors were of low relative influence in our study, it seems unlikely that anthropogenic habitat destruction is the basis for the high relative influence of human population density on the distribution of large-bodied reef fishes in this study. It should be noted that, with the exception of the two shark species, each of the chosen species do appear, in varying levels, in commercial and recreation catch data from Guam and the Commonwealth of the Northern Mariana Islands. While *L. bohar* is considered ciguatoxic in the Mariana Archipelago, and is therefore not commonly considered a “fishery target”, the species does appear—albeit in modest amounts—in both boat-based and shore-based creel survey data from Guam, as well as in commercial landings data, where it is classified under the the common name “Tagafi” and possibly as a component of the overall “snapper” grouping (unpublished data collected and processed by Guam Division of Aquatic Resources and provided to the authors via the WPacFIN program [94]).

Interestingly, overall biomass density of *Cheilinus undulatus*, an IUCN Red List species, was higher in the southern islands than in the north, with the highest densities found around the islands of Rota and Sarigan, which have moderate to low human population densities. Initially we thought this might be due to a higher number of smaller individuals in the southern islands compared with a lower number of larger individuals in the north. However, this does not appear to be the case, as few individuals of any size were sighted in the northern portion of the archipelago. Of the environmental factors evaluated in the present study, depth had the highest relative influence with higher occurrence of *C. undulatus* at deeper depths. However, the waters around the southern islands tend to be shallower than those in the north, so this does not explain our findings. It is possible that *C. undulatus* distribution reflects the availability of certain habitats or conditions conducive to this species at Rota and Sarigan that we failed to measure, or the lack of the same in the northern islands. *Cheilinus undulatus* is an IUCN Red List species and is, therefore, of high management importance. However, it should be noted that *C. undulatus* was observed on only 49 of our surveys compared to a minimum of 100 survey observations for each of the other taxonomic groups. Hence, our results may be an artifact of low sample size, may not be representative of true distribution patterns, and should be interpreted with caution. Further, targeted research on this low-density species is needed to fully resolve the factors affecting its distribution.

All *in situ* surveys likely influence their study subjects, causing fishes to either aggregate around or flee from the observers or instrument platform [95]. We feel that the towed-diver methods used in this study are not as prone to bias as other diver-based techniques [53]. However, it should be noted that the relative difference between remote and human-inhabited islands could be exaggerated if fishes aggregate around divers in remote areas and flee from divers in areas frequented by people. That being said, our findings are consistent with the preliminary findings of Schroeder et al. [28], statements such as those by the Western Pacific Regional Fishery Management Council [26] suggesting that many large bodied species “became rare on shallow reefs [around Guam] due to heavy fishing …”, and the substantial literature linking anthropogenic impacts to decreases in fish populations [27], [32], [34], [38], [47], [48], [80]–[82], [96]–[106]. Fish and shellfish dominate middens dated to the period of early human habitation in the Mariana Islands and those from early settlement sites contain the remains of a wide range of fishes from coastal reefs, lagoons, and deep water (Green 1997). During this time, fishing appears to have been concentrated on coral reef and lagoon species [107] and stable isotope data indicate that marine foods constituted nearly 40% of the prehistoric diet [108]. Shortcomings in fishery statistics make it virtually impossible to assess the total harvest of contemporary coral reef fisheries in the Mariana Archipelago [26]. While most fisheries are limited to nearshore areas off Saipan, Rota, and Tinian, the accuracy of reporting is suspect in many areas and virtually no information is available for the inshore subsistence and recreational fisheries. At least six of the northern islands have been commercially fished to some extent [26], and poaching by foreign vessels has been documented [46]. While poaching is difficult to quantify, it is a legitimate concern as poachers tend to target high-value, rare, or otherwise heavily fished resources [26] – qualities that describe many large-bodied reef fish populations.

The evidence that early human populations had at least some impact on the local reef environment and that contemporary anthropogenic impacts may be greatly underestimated should be to be taken into account when discussing relationships between reef fishes and their habitat, as it is likely that contemporary relationships that include human influences may not necessarily be representative of healthy let alone “pristine” systems. Nevertheless, contemporary fish-habitat relationships can reveal the importance of certain physical, oceanographic, and biological factors in structuring the distribution of large-bodied reef fishes. As we discuss below, these relationships between fishes and their environment can inform discussions of important or “essential” fish habitats and their roles in ecosystem-based management.

### Fish - Habitat Relationships

The Boosted Regression Tree analysis, based on species presence/absence, was able to assess the relative influence of each environmental factor adjusting for the impacts of each of the other variables. Human population density was still the most influential factor, but others appear to play a role (Figs. 4 & 5). In certain cases, such as with the two shark species *Carcharhinus amblyrhynchos* and *Triaenodon obesus*, a look at prominent pairwise interactions among variables shows that variables including depth and wave energy are only influential in areas with the lowest human population densities. While the absolute influence of each variable was relatively low in this highly variable data set, oceanographic factors such as depth, temperature, and proximity to deep water (quantified as distance to the 50-m depth contour) consistently the most influential of the factors we analyzed. In almost all cases, species occurrence increased in deeper waters and in areas of higher temperature. The effects of temperature and depth were synergistic with respect to total large-bodied reef fish biomass, with the effect of each being magnified by the other. Depth was particularly important for *Cheilinus undulatus*, which was only observed on surveys in greater than 15 m. *Caranx melampygus* and *Scarus rubroviolaceus* displayed an inverse relationship with respect to depth, which highlights their differing niches. Carnivorous C. melampygus were primarily found in deeper waters (>15 m) while herbivorous *S. rubroviolaceus* were primarily found in the shallower areas (<15 m) more conducive to algae growth. Proximity to deep water was particularly important for *Carcharhinus amblyrhynchos*, species of *Macolor*, *Caranx melampygus* and *Naso hexacanthus*, which are all found near escarpments or walls. These findings are consistent with those of Friedlander et al. [88] who found depth and proximity to the pelagic environment to predictably alter the structure of reef fish assemblages at Kingman Reef in the northern Line Islands. Specifically, these authors found that both abundance and biomass increased with proximity to the pelagic environment and with increasing depth on the forereef. Our findings are also consistent with those of Wetherbee et al. [109] and Papastamatiou et al. [110] who found that CPUE or the relative number of grey reef sharks (*Carcharhinus amblyrhynchos*) caught in Hawai'i generally increased with depth. Friedlander et al. [88] also found that apex predators, and the planktivorous snappers, on which they prey, were more abundant on the deeper forereef and reef walls, presumably because currents consistently deliver higher concentrations of plankton to these areas [111], [112]. These findings are consistent with the relationship we describe regarding the planktivorous species of *Macolor* and *Naso hexacanthus*. It is possible that the relationship between the apex predators (*Carcharhinus amblyrhynchos*, *Caranx melampygus*) and proximity to deep water is a secondary relationship related to the distribution of their prey species. This conclusion is consistent with Wetherbee et al. [109] and Papastamatiou et al. [110], who found that teleost fishes (e.g., holocentrids, monacanthids, and acanthurids) dominated the gut contents of *Carcharhinus amblyrhynchos* in the Hawaiian Islands.

*In situ* temperature also factored highly in the distribution of large-bodied reef fish, with overall biomass and the occurrence of many taxa positively correlated with temperature. In certain taxa including *Carcharhinus amblyrhynchos* and *Lutjanus bohar*, temperature showed high relative influence, but the relationship did not have a clear linear trend. In the case of *L. bohar*, there was an interaction between temperature and wave energy in which the effect of temperature was magnified in areas of high wave energy. The thermal environment of coral reefs is highly stable and thermal gradients can play a large role in life history and distribution both on and off coral reefs [113]–[116]; hence it is not surprising that fish react to temperature and that the relationship can be quite complex. Meyer et al. [19] found that short-term movements of *Aprion virescens* at Pearl and Hermes Reef in the NWHI were oriented to tidal cycles, with fish moving along the barrier reef on the flooding tide and returning on the ebbing tide, and tidal rhythmicity has been previously documented in acoustic monitoring studies of other fishes. We did not account for tidal cycle in our analysis, and it is possible that the importance of temperature is magnified by a correlation with tidal cycling. There may also be a relationship between temperature and primary productivity that would follow the north-south human population gradient. However, unlike remotely sensed mean sea surface temperature, *in situ* temperature measurements did not clearly correlate with other environmental gradients measured in this study (max ρ = 0.41 with % sand cover) and the difference in mean *in situ* temperature measurements between remote and populated sections of the archipelago (the strongest overall pattern) was only 0.4°C.

Topographic complexity, benthic habitat structure, and benthic cover had relatively little influence in our analysis of large-bodied reef fish. In contrast, these factors have a strong influence on the distribution and relative abundance of smaller reef fishes [5], [6], [8], [11], [89], [91], [117], [118]. It is likely that our estimates of topographic complexity, which are based on a six-point scale visually estimated by divers, are more subjective or variable than quantitative measures such as multibeam SONAR or LIDAR. These latter types of data might provide a better means for assessing this relationship, but, unfortunately, they are presently unavailable for these areas. Organisms interact with their environment at a range of scales, and the relative heterogeneity of the environment depends on the size of the individual [93]. It is possible that the resolution at which we measured these variables is not ideal for the large-bodied portion of the reef fish assemblage.

Benthic habitat structure appeared to be important for *Lutjanus bohar* and *Cheilinus undulates*, with higher occurrences of these species in areas characterized as “Rock/Boulder” and “Pavement” or “Aggregate Reef,” respectively. While a majority of species showed a positive relationship with benthic complexity, the relative influence of complexity was not high compared to the other measured variables. This may be because many large-bodied reef fishes are not as closely associated with small-scale benthic structures (but see Lindberg et al. [17], tend to have wider ranges, and interact with their environment in a more “coarse-grained” manner (sensu Levins [93]). It is also possible that we failed to measure habitat characteristics in sufficient detail or at the scales important to this portion of the assemblage. As noted above, the ability to incorporate high-resolution bathymetric information for large areas, such as that provided by multibeam SONAR or LIDAR technologies, would likely allow for a more detailed investigation of these relationships.

Fisheries management in coral reef ecosystems is moving towards ecosystem-based approaches with a priority placed on the identification of “essential fish habitat” – ‘those waters and substrate necessary to fish for spawning, feeding, breeding, and growth to maturity’ [7], [26]. However, logistical constraints have limited much of the previous research to small-bodied coral reef fishes and often to areas adjacent to human populations [8], [119]–[121]. As many fisheries tend to preferentially target large-bodied species and large individuals [48], [122], [123], determination of the relative importance of environmental factors for this segment of the assemblage is essential to improving fisheries and ecosystem-based management. Our results show that proximity to areas of high human population had the highest influence relative to the other environmental factors we measured and likely influences large-bodied reef fish assemblages in the Mariana Islands. Whether this is a result of fishing or other anthropogenic impacts for which we do not yet have accurate data is unknown, but extraction is commonly assumed to be the most important impact associated with human population centers [27], [80], [101], [124].

While the negative relationship with human population was the most important predictor variable in this study, the human population gradient was correlated with latitudinal and temperature gradients. Large-scale oceanographic factors such as depth, temperature, and proximity to deep water were also important. Shallow-water wave stress may be significant locally, but this is difficult to quantify accurately without bathymetry data. Large-scale habitat structure was also shown to be important for certain species. Conveniently, data on many of these factors may be easily obtained at large scales through remote sensing, allowing managers and researchers to develop predictive models delineating areas conducive to large-bodied reef fishes using methods similar to those outlined by Pittman et al. [12], De'ath [72] and Elith et al. [61]. Such information can be used in ecosystem-based spatial planning and management. For example, even for cases in which visual or other population survey data are lacking, areas likely capable of supporting species of particular concern can be identified as a areas for further research or consideration as marine protected areas. The reader will note that, as with other studies focusing on low density and patchily distributed assemblages, our models explain only a small portion of the overall deviance. There are likely many factors that contribute to the spatial and temporal distribution of individual species. Our aim has been to evaluate the relative influence of a specific suite of environmental variables that have been identified as important in previous studies. By gaining a better understanding of the relationships between large-bodied reef fishes and their environment, especially with those environmental variables that showed high relative influence, management agencies can better conserve large-bodied reef fish populations. While many of the environmental variables identified as important in this study cannot be directly manipulated, such as proximity to human population or distance to deep water, our results can highlight the importance of certain areas, such as remote and uninhabited islands with steep walls, that may serve as important refuges for these fishes and are therefore worthy of protection and monitoring. With appropriate caution, model predictions resulting from this kind of research can be used in an adaptive management framework to adjust the boundaries of existing marine protected areas that are not meeting their management objectives and to target further detailed research.
